# Perceptions of Healthcare Professionals Regarding the Cultural Food Practices of African Migrant Women During Pregnancy and Postpartum in Australia

**DOI:** 10.1111/mcn.70183

**Published:** 2026-04-17

**Authors:** Bolanle R. Olajide, Paige van der Pligt, Vidanka Vasilevski, Fiona H. McKay

**Affiliations:** ^1^ Institute for Health Transformation (IHT), School of Health and Social Development Deakin University Burwood Victoria Australia; ^2^ Department of Allied Health, School of Health Sciences Swinburne University of Technology Hawthorn Victoria Australia; ^3^ School of Health and Social Development Deakin University Burwood Victoria Australia; ^4^ Department of Nutrition and Dietetics Western Health Footscray Victoria Australia; ^5^ School of Nursing & Midwifery, Centre for Quality and Patient Safety Research, Institute for Health Transformation Deakin University Burwood Victoria Australia; ^6^ Western Health, St Albans Victoria Australia

**Keywords:** culture, food practices, healthcare provider, migrants, postpartum, pregnancy, qualitative research

## Abstract

In African societies, cultural food practices often restrict or prohibit the consumption of certain foods during pregnancy. While some of these practices persist after migration, how healthcare professionals (HCPs) perceive the influence of these practices on African migrant women's food practices in Australia remains unexplored. Understanding HCPs' perceptions of African migrant women's food practices will provide insights into how these practices are understood and addressed in clinical settings. This study examines HCPs' perceptions of the cultural food practices and nutrition behaviours of African migrant women during pregnancy and the postpartum period in Australia. Participants were recruited through convenience and snowball sampling. In‐depth semi‐structured interviews were conducted with 15 HCPs who had experience providing antenatal care to African migrant women in Australia. Thematic analysis was used to analyse the data. Three themes were identified: (1) HCPs' perceptions of women's cultural food practices, (2) challenges in delivering general healthy eating information, and (3) strategies for providing effective nutrition advice. Few HCPs were aware of the specific restrictive food practices among the African migrant women they support. HCPs perceived that the healthy eating information provided to these women was not always effective and culturally appropriate due to the constraints on consultation time and a lack of culturally appropriate resources. Participants expressed a need for in‐service education, tailored resources on African foods to enhance culturally appropriate care, and support for continuity of care. This study highlights the challenges HCPs face in providing culturally appropriate nutrition support to African migrant women during pregnancy and postpartum. Addressing these gaps through supportive training for HCPs and culturally tailored resources for women is needed.

## Summary

- HCPs recognised that culture influences African migrant women's food practices during pregnancy; however, most had limited awareness of specific culturally based food restrictions, particularly those related to meat intake.
- Limited consultation time, lack of culturally tailored nutrition resources, and insufficient culture‐specific training constrained HCPs' ability to deliver effective and culturally appropriate healthy eating information.
- HCPs identified continuity of care, access to dietitians, culturally tailored nutrition resources, and targeted professional development as key strategies to support more culturally responsive antenatal nutrition care for African migrant women.

## 1 Introduction

Culture plays a pivotal role in shaping dietary behaviours, influencing both the content and timing of an individual's food consumption. During pregnancy, these dietary behaviours become even more pronounced, with women's food choices often dictated by aversions and cravings associated with both pregnancy and cultural beliefs (Chakona and Shackleton 2019; Yalew et al. 2021). Research from South Africa and Ethiopia (Ramulondi et al. 2021; Tela et al. 2020; Wondimu et al. 2021) highlight the impact of cultural norms on the food practices of pregnant women living in Africa. These practices involve the avoidance of some foods due to concerns about congenital malformation, miscarriage, or unplanned caesarean section (Tela et al. 2020; Wondimu et al. 2021). Foods that are commonly avoided by pregnant women include animal products (such as meat and fish), fruits (such as pineapple and apple), and vegetables and legumes (such as potatoes, beans, and pumpkin) (Ramulondi et al. 2021; Wondimu et al. 2021). A review of the food choices and experiences of pregnant and postpartum migrant women from low‐ and middle‐income countries found that these culturally driven dietary practices often persist after migration (Olajide et al. 2024). While women may compensate by gaining nutrition from other foods, there are concerns that food avoidance, particularly when not guided by medical or nutrition professionals, could lead to potential macronutrient and micronutrient deficiencies during pregnancy (Chakona and Shackleton 2019).

Despite evidence that migrant women often continue with cultural food practices after migration (Ngongalah et al. 2023; Olajide et al. 2024), the process of dietary acculturation can influence how these practices are maintained or modified in host countries. Dietary acculturation refers to the gradual transition from familiar traditional foods to the eating patterns commonly available in high‐income Western settings (Osei‐Kwasi et al. 2023). Research with African migrant women in Canada (Blanchet et al. 2018) and Australia (Mude and Nyanhanda 2023) has reported increased consumption of Western foods such as fast foods and pizza, often attributed to the affordability and accessibility of these foods compared with traditional ingredients. Together, these findings illustrate how acculturation can shape the dietary transitions of African migrant women following migration.

Ensuring African migrant women receive and understand the health care information available to them is a challenge. Research suggests that the pregnancy nutrition information that sub‐Saharan African migrant women receive from HCPs in their new countries does not always suit their needs (Iradukunda and Poudel‐Tandukar 2021). Research with African migrant women in the United Kingdom and the United States (Iradukunda and Poudel‐Tandukar 2021; Ngongalah et al. 2021), found that despite reporting that they were receiving more pregnancy nutrition information from HCPs than they would expect in their home countries, the information was inaccessible. For example, women were instructed to consume specific nutrients beneficial for pregnancy, but they often struggled to identify which of their usual meals contained these nutrients (Ngongalah et al. 2021). In general, the information they received was considered too detailed, culturally inappropriate or difficult to navigate.

Compounding the challenges African migrant women face in understanding the advice provided by HCPs are the difficulties HCPs experience themselves in delivering maternity care to women from diverse cultural backgrounds (Akhavan 2012; Oscarsson and Stevenson‐Ågren 2020; Shorey et al. 2021; Söderström et al. 2022). Language barriers and cultural differences have been identified by HCPs, including midwives, nurse practitioners, and obstetrician and gynaecologists as challenges in conveying health information effectively (Akhavan 2012; Ng and Newbold 2011; Shorey et al. 2021). These challenges underscore the complexities HCPs encounter when providing maternity care to migrant women with diverse cultural backgrounds.

While much of the existing research has focused on African migrant women's perspectives (Ngongalah et al. 2023; Olajide et al. 2025), far less is known about how HCPs understand and respond to these cultural food practices. Exploring HCPs' perceptions is important because their interpretations of women's dietary behaviours influence the type of nutrition advice and support offered during pregnancy. Understanding these perspectives can help identify potential mismatches between women's cultural expectations and the care they receive and may highlight areas where communication or support strategies need strengthening. Examining HCPs' views therefore complements research centred on women's experiences and contributes to developing more culturally responsive nutrition care within maternity services.

Given the important role of antenatal HCPs in supporting the best maternal health outcomes and fostering culturally sensitive care, the aim of this research is to explore their perceptions of the cultural food practices and nutrition behaviours of African migrant women during the perinatal period. The rapid increase in the African migrant population in Australia, from approximately 338,000 in 2011 to 495,000 in 2021 (Australian Bureau of Statistics 2011, 2021), underscores the need for a better understanding of the health needs of this growing population. This is particularly critical as African‐born women living in Australia experience a higher risk of adverse maternal outcomes, including very low birthweight, preterm birth, and severe postpartum haemorrhage, compared to Australian‐born women (Belihu et al. 2016; Eslier et al. 2023).

## 2 Methods

A qualitative approach was employed to explore HCPs' perceptions of the cultural food practices and nutrition behaviours of African migrant women through in‐depth semi‐structured interviews. In‐depth semi‐structured interviews are commonly used to collect data in qualitative research, aiming to gather insights from individuals with personal experiences and perceptions related to the subject being studied (DeJonckheere and Vaughn 2019).

### 2.1 Settings and Participants

Eligible participants included HCPs who had experience of providing antenatal or nutrition care to African migrant pregnant women in Australia. Experience providing antenatal care to African migrant women was defined broadly and did not require a minimum duration or number of encounters. Potential participants included midwives, general practitioners, dietitians, and obstetricians working in health settings such as hospitals and community health centres. Given that participants were required to have experience caring for African migrant women, this study employed a purposive sampling approach to recruit eligible HCPs. Recruitment was conducted using convenience and snowball sampling methods. Recruitment commenced in February 2024 and continued concurrently with data collection until interviews were completed. A recruitment flyer was distributed through professional networks, contacts with hospitals, and posted on social media platforms (LinkedIn, Twitter/X, and Facebook). The recruitment flyer contained a research description, a QR code, and the researcher's email address. Interested HCPs could either scan the QR code or email directly to access a Qualtrics survey.

The first author reviewed a list of public and private hospitals in Australia, and contact details were extracted from each hospital's website. Following this step, approximately twenty hospitals were contacted via phone call or email, and the recruitment flyer was shared with these hospitals for distribution among relevant staff. Recruitment was also conducted through professional associations, including the Australian College of Midwives' ‘midwife chat’ forum, to target midwives with relevant experience across Australia. In addition, a paid social media campaign was conducted to target HCPs in Australia, and the advertisement included a direct link to the Qualtrics survey. Participants completed a Qualtrics survey to confirm their eligibility and provide their contact details for an interview. After agreeing to participate, HCPs were sent an email to schedule an interview. Those who did not respond to the initial email were contacted up to three times.

### 2.2 Data Collection

Data were collected via in‐depth individual interviews between March and August 2024. Interviews were conducted via Zoom or by phone. The interviews were informed by a semi‐structured interview guide designed to elicit information regarding the experiences of HCPs caring for pregnant African migrant women (see Supporting file 1). The interview guide was developed based on the study's aims and informed by existing literature on cultural food practices and migration‐related dietary experiences. Interviews were between 30 and 55 min in duration and were audio recorded. The recorded interviews were transcribed verbatim before analysis. All data were collected by a researcher experienced in qualitative interviewing. Interviewing continued until no new themes were identified within the participating HCP group, which occurred after 13 interviews. However, two additional interviews were conducted with participants who had previously made a time and needed to reschedule. Member checking was used to enhance rigour by allowing participants to review and validate their full interview transcripts (Birt et al. 2016). Full transcript review was chosen to ensure participants had the opportunity to check the complete accuracy of their narrative, which is a recognised method for enhancing credibility in qualitative research. Two HCPs engaged in the member checking process; both fully agreed with the transcriptions without requesting changes. Participants were compensated for their time.

### 2.3 Data Analysis

Data were coded using NVivo 14, and the analysis involved a six‐phase reflexive thematic analysis process (Braun and Clarke 2022). Reflexive thematic analysis is a qualitative approach that encourages researchers to acknowledge their subjectivity, prompting reflection on their assumptions and practices, and how these factors can influence data analysis (Braun and Clarke 2022). An inductive approach to analysis was undertaken by identifying themes from participants' narratives. The analysis included data familiarisation, coding, theme exploration, theme review, definition and naming of themes, and final documentation. Three authors independently coded one transcript each, while the first author also coded the same transcript to support a consistent approach to the analysis. Following this, the first author coded and analysed the remaining data. The resulting codes and themes were subsequently discussed among the authors to ensure a shared understanding of the data and to further enhance the rigour of the analysis. No discrepancies in coding or theme development were identified during this process. Transferability was enhanced through a detailed description of the study methods. Participants' quotes are denoted as ‘P’, followed by the participant number, role, country of origin, and years of experience providing antenatal care to African women in Australia.

### 2.4 Ethical Statement

Ethics approval was obtained from the Deakin University Human Research Ethics Committee (approval number: 2023‐331), granted on November 2, 2023. Informed consent was obtained from all participants prior to study participation, including consent to the publication of their anonymised responses. The study was conducted in accordance with the National Statement on Ethical Conduct in Human Research (National Health and Medical Research Council, Australian Research Council, & Universities Australia 2007).

### 2.5 Positionality

The researchers' positionality is integral to this study. The first author, a female PhD student from an African country with a background in nutrition and dietetics, conducted the recruitment, interviews, and analysis. Her cultural background may have shaped how she engaged with participants and interpreted discussions about cultural food practices. The co‐authors (van der Pligt, Vasilevski, and McKay) are academics and practicing professionals specialising in fields related to pregnancy, including dietetics, food security, psychology, and maternal and child health. Their disciplinary perspectives informed how codes and themes were refined, contributing to broader interpretation of the data. All authors have experience in qualitative research, and none had prior relationships with participants.

## 3 Results

### 3.1 General Characteristics of the Sample

Of the 40 individuals who provided informed consent, 20 met the eligibility criteria and were invited to participate. Of the eligible participants, 15 completed in‐depth semi‐structured interviews. Reasons for non‐participation included a busy schedule (*n* = 3) and non‐responsiveness to follow‐up emails (*n* = 2). Most HCPs (*n* = 13) were midwives, two of whom held additional qualifications, the remaining two HCPs were obstetrician and gynaecologists. The average age of participants was 41.6 years (range: 24–63). For further demographic details, see Table 1.

**Table 1:** Demographic details of participants (*n* = 15).

Demographic details	*n*
Place of birth	
Australia	9
Africa^a^	5
Asia^b^	1
Age	
20–30	2
31–41	6
42–52	5
53–63	2
Sex	
Female	13
Male	2
HCPs category	
Midwife	11
Midwife and nutritionist	1
Midwife and nurse practitioner	1
Obstetrician and gynaecologists	2
Years of experience providing care for African migrant women	
1–5	9
6–10	2
11–15	2
≥ 16	2

^a^ Nigeria, Ghana, Kenya, and Zambia.

^b^ Sri Lanka.

Three main themes and two sub‐themes were identified in the analysis. These themes related to HCPs' perceptions of women's cultural food practices, the challenges they face in delivering general healthy eating information, and the strategies they identified for addressing these challenges. In these results, African migrant women are referred to as ‘women’.

### 3.2 Theme 1: HCPs' Perceptions of Women's Cultural Food Practices

Participants discussed their perceptions of women's food practices during pregnancy and the postpartum period in Australia. Two sub‐themes emerged: (1) HCPs' perceptions of women's adherence to cultural food practices and (2) the influences shaping these practices.

#### 3.2.1 1a: HCPs' Perceptions of Women's Adherence to Cultural Food Practices

Participants discussed the extent to which they perceived women to adhere to their cultural food practices after migrating to Australia. Most participants stated that they observed women continuing their traditional dietary practices for a number of reasons.

…just because they have become pregnant doesn't mean they're going to change their whole diet … if they normally eat like a traditional diet, then they'll probably continue.

(P4, Midwife, Australia, 1 year)

A few HCPs observed that Somali women adhered more strictly to their traditions than other African women, who more readily adopted a Western diet.

… Somali women tend to maintain their Somali culture, quite strongly… Where[as] some women from other communities like West Africa were somewhat more wanting to adapt to a Western culture.

(P13, Midwife, Australia, 8 years)

Several participants considered adherence to cultural food practices as related to generational differences. They noted that younger women were less likely to follow traditional practices, often favouring convenience foods, which they perceived could lead to health issues such as gestational diabetes and overweight during pregnancy.

… the younger generation…don't necessarily want to eat the traditional food…they tend to eat too much fast food…that's why we're seeing more overweight pregnancies and more GDM in the demographic.

(P12, Midwife, Australia, 16 years)

The duration of residence in Australia was also considered influential in adherence to cultural food practices from HCP's perspectives. Participants observed that recently arrived women were more likely to adhere to traditional practices, while those who had lived in Australia for longer were more likely to adopt Western dietary behaviours.

People that had been in Australia for less amount of time… revert back to…traditional cultural practice as opposed to people…growing up…in Australia…open to the Western culture.

(P10, Midwife, Australia, 25 years)

The HCPs described the range of cultural food practices the women they care for engage with. These practices include restricting certain foods or consuming others and ‘eating for two’. Fewer than half of the HCPs (*n* = 5) were aware of food restrictions specifically during pregnancy. However, of importance is that four of these HCPs were of African heritage. The remaining HCPs were unaware of the practice of culturally related dietary restrictions, stating that it was not a part of their education.

I've never come across that [food restriction] in nutrition or in midwifery studies… we missed a whole bunch of stuff.

(P9, Midwife, Australia, 2 years)

The HCPs who cared for women with these practices reported a range of foods that were commonly restricted, including eggs, some fish (especially fish with no scale), snail, certain meat (beef, duck, and pork), pineapple, and certain seafoods (prawns, octopus, and salmon). These HCPs further reported that the restrictions were perceived to be rooted in cultural beliefs rather than what are considered healthy eating guidelines in Australia. One HCP described their experience of advising women on the nutritional benefits of these commonly restricted foods.

…some cultures where women were told… not to eat pineapple and red meat. I… explain to them the nutritional benefits.

(P7, Obstetrician and Gynaecologist, Kenya, 2 years)

Others shared that they suggested substitutes to restricted foods to ensure women's adequate nutrition. For instance, replacing a restricted source of protein with an alternative.

…if they are avoiding something like snail, I then advise them, chicken and meat they can eat.

(P2, Midwife, Nigeria, 3 years)

Few HCPs reported that they observed women to restrict meat intake during pregnancy. However, some HCPs observed that Nigerian women increased their intake of meals comprised of meat and oily carbohydrates when they made postnatal visits. They noted that these meals were consumed as they were traditionally perceived to support breastfeeding during the postpartum period.

…the Nigerian women… [consume] so much meat…this is really oily…the oily rice…that was all postnatal to support breastfeeding…

(P11, Midwife, Australia, 8 years)

Only a few HCPs observed that women engaged in the practice of ‘eating for two’ during pregnancy, which they perceived as a cultural food practice of African communities. This finding differs from studies in other high‐income settings, where the ‘eating for two’ belief has been more commonly reported among African migrant women.

…in pregnancy you can eat for twotwo…… They [African women] said that they need to eat more food, whereas they don't need that.

(P15, Midwife, Australia, 2 years)

The practice of consuming or avoiding hot or cold foods was also observed by HCPs. Two HCPs connected this to a shared cultural practice, similar to other cultural traditions, where the warmth helps with blood circulation and fat burning post‐childbirth.

…no ice or cold foods…African women have got a concept of keeping the body warm…to maintain bodily function… which is the Chinese medicine and…Indian Ayurvedic practise.

(P11, Midwife, Australia, 8 years)

#### 3.2.2 1b: HCPs' Perceptions of the Influences on Women's Cultural Food Practices

Participants discussed the influences of culture and tradition on women's food practices. Most HCPs considered cultural norms to be a major influence on women's food choices. Cultural norms were considered those practices that are passed through generations and are rooted in the communities where women were raised. Few HCPs presumed that women followed these practices because they observed other women in their communities doing the same.

…just what they [African women] have seen been done in their communities…while they are growing up, they probably do the same…

(P2, Midwife, Nigeria, 3 years)

Family influence was frequently mentioned as shaping women's food practices during pregnancy and after birth. HCPs observed that families often provided guidance on what to eat and, in some cases, brought traditional foods to support women's recovery postpartum.

The biggest influence that I've noticed as a midwife…is that everyone's family is bringing them the traditional foods to nourish them and replenish their blood.

(P9, Midwife, Australia, 2 years)

Conversely, a minority of HCPs considered that the absence of family members living with women gave them more freedom to make independent food choices.

…when…they are not directly living with their family… it becomes easier for them to break from such practises…

(P7, Obstetrician and Gynaecologist, Kenya, 2 years)

Friends were also identified as having an influence on women's food practices. Some HCPs mentioned that women often observed the practices of friends and sought confirmation from them about the accuracy of the information they received about food choices.

… my friend did this. My friend said this…Is that accurate?…Friends are telling them like …having too much red meat isn't good for you…

(P15, Midwife, Australia, 2 years)

### 3.3 Theme 2: Challenges in Delivering General Healthy Eating Information

As part of their role in seeing pregnant women and providing general healthy eating information, HCPs reported that women were open to discussing food and receiving nutrition guidance during pregnancy. However, they also noted that they were not well‐equipped to deliver such information. While they recognised the importance of providing healthy eating information, they identified several barriers to effective delivery and cultural appropriateness. These included language difficulties, limited consultation time, a lack of awareness of culturally tailored resources in hospitals, and insufficient cultural sensitivity training. Although HCPs acknowledged that they are not always responsible for delivering culture‐specific nutrition advice, they emphasised the importance of ensuring women have access to dietitians or resources that could offer more personalised support.

If it were possible for all these women…get an opportunity to see a [nutrition professional], not just [only those considered high risk or with metabolic issues] …be had with [dietitians].

(P7, Obstetrician and Gynaecologist, Kenya, 2 years)

Language barriers were considered a major challenge in delivering general healthy eating information. While nearly all HCPs confirmed the availability and use of interpreter services when required, some noted that these services were not always accessible. In cases where an interpreter was not available, HCPs said that the women's partners acted as an interpreter, which they considered a challenge. They expressed concerns about the accuracy of the information conveyed through this approach.

…not always having an interpreter if needed…using [the] husband as an interpreter, you'll not get the full conversation… don't know what's being absorbed.

(P8, Midwife, Nigeria, 3 years)

In addition to language barriers, limited time and lack of culturally tailored resources in hospitals were identified as challenges to delivering culturally appropriate healthy eating information to women. Some HCPs reported that they often did not have enough time to explain healthy eating to women or go through the hospital‐provided general healthy eating resources in detail. As a result, nutrition became a lower priority during appointments.

…if you're lucky, 20 minutes for a booking in visit…time constraint is the biggest thing. You have so many other things to cover than unfortunately, nutrition.

(P3, Midwife, Ghana, 12 years)

A minority of HCPs who worked with other cultural groups stated that these groups had nutrition materials specifically designed for them. However, they reported that they were unaware if any African specific resources were available for women.

…for other cultures we have like infographic pictures, like for Indian women. Like what to swap for white rice, with pictures…we don't have one for African women.

(P4, Midwife, Australia, 1 year)

Opportunities for cultural sensitivity training were also identified by HCPs as a way to delivering more effective healthy eating information to women. Some midwives expressed concerns about the lack of training specific to individual cultures.

We don't receive specific training…in terms of providing culturally sensitive care to specific culture, we just sort of learn as we go on the job.

(P6, Midwife, Australia, 5 years)

### 3.4 Theme 3: Strategies for Providing Effective Nutrition Advice

In response to the challenges that HCPs identified in providing general healthy eating information to women, all HCPs suggested strategies to address these issues. These strategies included education given by a dietitian or African HCPs, culturally tailored resources to be available in the hospital, continuity of care, and employing African or bicultural staff in hospitals.

Nearly all HCPs expressed a need for in‐service education or training regarding cultural food practices of women, emphasising the importance of expanding their knowledge in this area. They suggested various ways in which this could be delivered including face‐to‐face and online formats.

… having access to education…someone come in like in‐service and speak to us …there's not much information in terms of… food practices for African women…

(P6, Midwife, Australia, 5 years)

Most HCPs suggested that the availability of culturally tailored resources specific to African cultures would enhance their ability to provide culturally appropriate nutrition information to women. The suggested resources included pamphlets, food guides incorporating African foods, and online materials. Participants expressed a preference for resources that highlight foods specific to women's countries. They further emphasised that such resources should be co‐developed by people within these communities.

…the ability to pull a resource that says this lady is from this country…would happily eat this…

(P12, Midwife, Australia, 16 years)

Some HCPs suggested that one way to address time constraints is through a continuity of care model. They stated that having women see the same midwives throughout pregnancy and into the postpartum period could improve health outcomes and foster a sense of trust.

…if there is continuity of care instead of seeing different person each time…improve outcomes for women in pregnancy…midwife doesn't have to be African…

(P11, Midwife, Australia, 8 years)

The importance of employing staff with African heritage or knowledge was identified as another potential strategy for delivering effective nutrition information to women. The HCPs of non‐African heritage highlighted that they had observed women tended to trust nutrition advice provided by colleagues of African background, rather than those by non‐African HCPs. Based on this observation, they suggested employing African or bicultural staff in hospitals.

…colleague from a similar background [is present]… give nutrition advice to them [women], they usually develop more trust… important to have African women working in roles like dietitians in the hospital…

(P1, Midwife, Australia, 13 years)

## 4 Discussion

This study explored HCPs' perceptions of the cultural food practices and nutrition behaviours of African migrant women during pregnancy and the postpartum period in Australia. A key finding was that HCPs recognised cultural norms as influential in shaping women's food practices, but they had limited awareness of specific pregnancy food restrictions. This gap in knowledge may have implications for both maternal and foetal health outcomes, and the relationship between HCPs and women. The study identified several barriers to delivering effective nutrition information and suggested strategies to address these challenges. The HCPs also expressed concern that the nutrition information they provided may not be appropriately tailored to these women. These findings highlight the need for increased access to dietitians during pregnancy and postpartum, and for the development of culture‐specific, tailored resources that HCPs can use to support women. Such resources could improve the delivery of important nutrition information to African migrant women.

While previous studies with pregnant African migrant women in the United States and United Kingdom have highlighted some culturally inappropriate nutrition information provided by HCPs (Iradukunda and Poudel‐Tandukar 2021; Ngongalah et al. 2021), these studies focused solely on women's perceptions. The current study builds on this by capturing HCPs' perceptions. Consistent with previous research, the current study found that HCPs face barriers providing antenatal care to migrant women (Andersson et al. 2021; Hughson et al. 2018; Olcoń et al. 2023; Söderström et al. 2022). The findings also revealed that HCPs encounter several challenges providing effective and culturally appropriate healthy eating guidance. These challenges may be linked to systemic healthcare limitations and factors beyond their control, such as lack of awareness of African women's food practices, limited access to tailored resources, short consultation times, and the absence of culture‐specific nutrition training in professional development programs (Arrish et al. 2017). Collectively, these issues point to broader organisational and structural constraints within the health system, illustrating that culturally competent care is shaped not only by individual provider knowledge but also by the institutional conditions under which care is delivered.

Participants in this study emphasised the importance of nutrition advice being given to pregnant women but reported multiple barriers to the delivery of effective and culturally appropriate healthy eating information. A key challenge identified was limited consultation time, which restricted their ability to discuss nutrition related concerns. This finding is consistent with research with Australian midwives, who reported that time constraints hindered their ability to provide quality care to Somali women during hospital visits (Andersson et al. 2021). Interpreting this finding through a cultural competence lens provides additional explanatory depth. Cultural competence frameworks emphasise that effective cross‐cultural care depends not only on providers' knowledge but also on organisational conditions that support culturally responsive interactions (Cross 1989). Within this context, system‐level factors, such as limited consultation time can constrain HCPs' opportunities to engage in meaningful, culturally informed nutrition discussions. Addressing these limitations may require alternative models of care, such as the continuity of care approach. Continuity of care has been associated with positive experiences of care, the development of trusting relationships, and more personalised support among migrant women (Billett et al. 2022; Fair et al. 2020). Within such a model, midwives may have greater opportunities to provide culturally appropriate healthy eating advice. This may be effective when supported by dietitian‐led training and access to culture‐specific resources.

The lack of accessible culturally tailored nutrition resources for African women was another challenge expressed by HCPs. This aligns with the work of Ekong (2022), who found that the lack of tailored resources made it difficult for African women to access culturally relevant and reliable nutrition information. From a systems perspective, this absence of resources reflects structural inequalities in how health information is developed and prioritised (Betancourt et al. 2003; Truong et al. 2014). The absence of such resources not only challenges HCPs but places African migrant women at a disadvantage when seeking to access culturally appropriate nutrition guidance. This gap could have long‐term health consequences, as women may continue to rely on conflicting or inadequate information, potentially leading to poor dietary choices that affect maternal and foetal health. This concern is supported by evidence from a review of African migrant women residing in high‐income countries, which reported that women often had lower micronutrient intake and made dietary adjustments, including increased consumption of processed foods, which may not meet pregnancy nutritional requirements (Ngongalah et al. 2018). This is particularly concerning given that, among migrant populations in Australia, African women are at increased risk of having a low birth weight baby compared with Australian‐born women (Mozooni et al. 2023). Similarly, African‐born women have been found to be at greater risk of having a very preterm birth and a higher likelihood of being diagnosed with gestational diabetes compared with Australian‐born women (Australian Institute of Health and Welfare 2024; Belihu et al. 2016). To promote inclusive care, culturally tailored nutrition resources that support both HCPs and African women are needed. Notably, HCPs in this study said such resources should be co‐designed with African women to ensure their cultural relevance. In this context, co‐design could involve engaging African migrant women, community representatives, and HCPs through structured workshops to identify priority nutrition topics, culturally relevant foods, and preferred resource formats, while ensuring alignment with existing clinical guidelines. This underscores the importance of engaging migrant communities in the development of health education materials (Bartlett and Boyle 2022).

Cultural sensitivity was highlighted as one way of delivering effective care. Cultural sensitivity is a crucial component of high‐quality healthcare, enabling HCPs to build effective relationships with individuals from diverse backgrounds (Claeys et al. 2021). Participants specifically identified that professional development tailored to African women's culture could support their practice. Providing HCPs with adequate cultural sensitivity training and greater awareness of African cultural beliefs surrounding pregnancy could equip them to offer culturally appropriate, evidence‐based nutrition guidance. Yet, cultural competence frameworks emphasise that training alone is insufficient; competence also requires institutional support, adequate resourcing, and ongoing reflection on how biases and assumptions shape care delivery (Betancourt et al. 2003; Jongen et al. 2018). When accompanied by broader system‐level support, such training may improve communication, enhance trust between HCPs and African migrant women, and ultimately lead to better maternal and infant health outcomes (Claeys et al. 2021). Without this combined approach, HCPs may remain limited in their ability to provide culturally responsive care, thereby perpetuating health disparities in minority groups (Vella et al. 2022).

The perceptions of HCPs regarding the specific cultural food practices of African migrant women, such as food restrictions, revealed a limited awareness of these practices. Food restrictions, a common cultural practice observed by African women during pregnancy in various African countries (Ramulondi et al. 2021; Wondimu et al. 2021), often persist after migration (Chebet 2021; Ngongalah et al. 2023). This study found that the majority of HCPs were unaware of or had not observed these practices among the women they cared for. Only a few were aware of specific food restrictions observed during pregnancy. The limited awareness, particularly of red meat restrictions is concerning, as it may contribute to an increased risk of iron‐deficiency anaemia (Mohammed et al. 2019; Twum‐Dei et al. 2024). Although Australia‐specific data on iron‐deficiency anaemia among African‐born pregnant women are lacking, the potential nutritional implications underscore the importance of culturally informed nutrition discussions between HCPs and women. Iron‐deficiency anaemia is prevalent among women of African heritage and has substantial effects on pregnancy outcomes (Fite et al. 2021; Zegeye et al. 2021). Culturally relevant nutrition education for HCPs can better equip them to offer informed guidance that aligns with the nutritional needs of pregnant African migrant women.

Healthcare providers' interpretations of women's dietary choices also reflected how they perceived women adjusting to the Australian food environment. Some HCPs associated adherence to cultural foods with recent migration, family influence, or strong cultural ties, whereas younger women were described as more inclined to choose convenience foods. These perceptions align with acculturation theory, which proposes that migrants adapt their food practices in different ways depending on factors such as length of residence and social networks (Berry 1997; Satia 2010). While these interpretations reflect HCPs' viewpoints rather than women's own accounts, applying an acculturation lens helps illuminate how dietary transitions are shaped not only by cultural identity but also by exposure to new food environments. Recognising these varied acculturation pathways can help maternity services tailor nutrition discussions more appropriately, avoiding one‐size‐fits‐all assumptions and supporting women whose dietary transitions may increase nutritional vulnerability during pregnancy.

This study contributes new insights into how organisational, cultural, and systemic factors intersect to influence HCPs' ability to deliver culturally appropriate healthy eating information. By identifying gaps in training, resource availability, and system‐level support, the findings highlight opportunities to strengthen cultural competence within maternity services. They also point to the need for stronger support for culturally appropriate nutrition education during pregnancy. Strengthening cultural sensitivity training, integrating tailored nutrition resources and improving access to dietetic management may contribute to more culturally inclusive healthcare. The development of culturally appropriate resources and training programs may help to ensure equitable access to quality antenatal nutrition guidance for African women in Australia. This may be achieved by collaborating with African women and community members to ensure that resources and training programs reflect their cultural perspectives, needs, and experiences.

### 4.1 Strengths and Limitations

To our knowledge, this is the only study exploring HCPs' perceptions of African migrant women's food practices during pregnancy and the postpartum period in Australia. These findings enhance understanding of the challenges HCPs face in delivering effective and culturally appropriate nutrition information and suggest strategies to improve it. A key strength of this study is the use of individual interviews with HCPs who have varied experience of providing antenatal care to African migrant pregnant women. The qualitative approach provided rich, in‐depth information. However, the sample was predominantly composed of midwives, with limited representation from dietitians and other nutrition specialists. This reflects the professional composition of the participants who were available and responsive during the recruitment period, as HCPs can be a hard to reach population, despite efforts to recruit across multiple healthcare disciplines. As a result, the findings primarily reflect midwives' perceptions and may not capture the full breadth of nutrition‐specific expertise involved in antenatal care. It is therefore important to acknowledge that the perspectives of other professionals who are engaged with the care of pregnant women, such as dietitians, may differ. Future research should purposively include HCPs from diverse fields, particularly dietitians to broaden understanding and inform culturally appropriate nutrition and dietetic care during pregnancy.

## 5 Conclusion

This study highlights a gap in HCPs' awareness of food‐related cultural practices among African migrant women. The limited awareness of potential food restrictions observed during pregnancy can impact maternal nutrition and health. The challenges identified in delivering culturally appropriate nutrition guidance underscore the need for systemic changes in healthcare training and resources ideally developed with dietitians. Addressing the strategies suggested in this study is crucial, particularly the development of culturally tailored resources, which would benefit both African migrant women and HCPs. Culturally responsive healthcare plays a vital role in reducing health disparities and promoting more equitable maternal health services. Future research should include dietitians to explore their perceptions of the cultural food practices of African migrant women, given their specialised role in providing nutrition guidance.

## Author Contributions

All authors (Bolanle R. Olajide, Paige van der Pligt, Vidanka Vasilevski, and Fiona H. McKay) made substantial contributions to the conception, study design, and data analysis. Bolanle R. Olajide conducted all interviews, drafted the initial manuscript, and revised subsequent versions with feedback from co‐authors. Paige van der Pligt, Vidanka Vasilevski, and Fiona H. McKay supervised the study and critically reviewed the manuscript. All authors read and approved the final draft prior to submission.

## Conflicts of Interest

The authors declare no conflicts of interest.

## Supporting information

Supplementary file 1.

## Data Availability

The authors confirm that the data supporting the findings of this study are available within the article. Further inquiries can be directed to the corresponding author.
