# Supplementary material for: Perceptions of Healthcare Professionals Regarding the Cultural Food Practices of African Migrant Women During Pregnancy and Postpartum in Australia
Source: Matern Child Nutr. 2026 Apr 17;22(2):e70183. doi: 10.1111/mcn.70183 (PMC13087512; doi:10.1111/mcn.70183)
Supplement: Supplementary file 1 [file MCN-22-e70183-s001.docx]

**Interview Guide**

**Exploring the cultural food practices and influences of African migrant women during pregnancy in Australia.**

*Thank you for taking the time to talk to me today.*

*The aim of this research is to explore healthcare professionals' understanding of the influence of cultural food practices among African migrant women and their nutrition-seeking behaviours during pregnancy in Australia.*

*I believe that you have read and consented to participate in this research, but I would like you to go through the consent form again and check you are happy to be part of the research.*

*There are no right or wrong answers, and your opinions, ideas, and responses are valuable. Inform me of any queries, breaks, or if you wish to terminate the interview.*

*Do you have any questions before we begin?*

That’s great, are you happy to continue?

**OPENING QUESTIONS**

1. What is your current role?
2. How long have you been in this role?
3. How many women from African communities did you provide healthcare services to in a week/month?

**KEY QUESTIONS**

**Nutrition-seeking behaviour of pregnant African migrant**

1. What are your experiences so far in providing care for pregnant African women?
2. In this role, can you describe how or when African women ever ask advice regarding food and nutrition during pregnancy?
3. If yes, what approach do you use to offer nutrition advice to pregnant African women?

*(Prompt: Is this approach different from the advice provided to other groups?)*

1. In what format is this advice presented?

*(Prompts: Pamphlets, booklets, brochures, face-to-face contact?)*

1. Do you think pregnant African women follow the nutrition advice you give them? Why did you think so?

*(Prompts: culturally appropriate, difficult to understand?)*

1. What are challenges you had trying to communicate nutrition information to pregnant African women? How?

*(Prompts: cultural beliefs, language barrier, information conflict?)*

*We are about halfway through the interview. Are you okay to continue? Great.*

**Food practices of pregnant African migrant**

1. What is your understanding of the influence of culture or tradition as regards the food practices of African women during pregnancy?
2. Can you describe some of the food practices that African migrant women have engaged in during pregnancy based on your experience?

*(Prompts: restriction of foods, reduction in food intake, food taboos?)*

1. Do you think these food practices are beneficial to the mother and the fetus? How?
2. What factors do you think might influence the food practices of pregnant African women?

*(Prompts: Social, cultural, acculturation, economic factors?)*

1. What are the meanings that African women attached to their food practices during pregnancy or postpartum?

*(Prompts: hot foods, cold foods, good foods, bad foods?)*

1. What might help you to understand African women’s food practices during pregnancy better?

*We are nearly at the end of the interview. Are you happy to continue?*

1. How do you think pregnant African women can be supported to achieve optimal nutrition during this crucial period?
2. How could you be supported in your role to provide care that incorporates African women’s cultural food needs and expectations during pregnancy?

**CONCLUDING STATEMENTS AND ANY OTHER QUESTIONS**

We have covered a lot of different things today, but is there anything else that you would like to add?

What would you like me to take away from this interview?

Audio turned off.

Thank you for taking the time to talk with me today. I would like to confirm again if you would like to review a copy of the transcript and a summary of the findings.
